# Optimal Designs for Nonlinear Mixed-effects Models Using Competitive Swarm Optimizer with Mutated Agents

**DOI:** 10.21203/rs.3.rs-3389537/v1

**Published:** 2023-10-05

**Authors:** Elvis Han Cui, Zizhao Zhang, Weng Kee Wong

**Affiliations:** 1 Deaprtment of Biostatistics, UCLA, 650 Charles E Young Dr S, Los Angeles, 90095, CA, U.S.A.; 2 Alibaba Group, 969 West Wen Yi Road, Yuhang District, Hangzhou, 311121, Zhejiang, China

**Keywords:** Bayesian-optimal design, c-optimality, equivalence theorem, fractional polynomial, random effects, sensitivity plot

## Abstract

Nature-inspired meta-heuristic algorithms are increasingly used in many disciplines to tackle challenging optimization problems. Our focus is to apply a newly proposed nature-inspired meta-heuristics algorithm called CSO-MA to solve challenging design problems in biosciences and demonstrate its flexibility to find various types of optimal approximate or exact designs for nonlinear mixed models with one or several interacting factors and with or without random effects. We show that CSO-MA is efficient and can frequently outperform other algorithms either in terms of speed or accuracy. The algorithm, like other meta-heuristic algorithms, is free of technical assumptions and flexible in that it can incorporate cost structure or multiple user-specified constraints, such as, a fixed number of measurements per subject in a longitudinal study. When possible, we confirm some of the CSO-MA generated designs are optimal with theory by developing theory-based innovative plots. Our applications include searching optimal designs to estimate (i) parameters in mixed nonlinear models with correlated random effects, (ii) a function of parameters for a count model in a dose combination study, and (iii) parameters in a HIV dynamic model. In each case, we show the advantages of using a meta-heuristic approach to solve the optimization problem, and the added benefits of the generated designs.

## 1 Introduction

Meta-heuristics, and in particular, nature-inspired meta-heuristics, is increasingly used to tackle complex optimization problems in the real world that traditional algorithms cannot solve. They include high dimensional constrained nonlinear optimization problems, multiple-objective optimal design problems, or problems that do not have an explicit objective function to optimize. Meta-heuristics is now frequently used across disciplines to solve hard to optimize problems. For example, it is used widely in artificial intelligence [1] and during the pandemic, meta-heuristics was implemented in different ways to detect, track and predict COVID-19 infection rates. For example, [2] used meta-heuristics to develop cheap and fast diagnostic tools for detecting people infected with COVID19 to reduce the pressure on healthcare systems. Another example is [3], who created a discrete gaining-sharing knowledge-based meta-heuristic algorithm and derived an optimal disinfection scheduling strategies for cleaning public places during the pandemic. [4] reviewed numerous applications of meta-heuristics to study COVID19. Meta-heuristics has also made significant contributions in using medical images to classify various diseases and predict disease progression in lung cancer, cardiovascular diseases and renal failure [5, 6]. [7] discussed concrete applications of meta-heuristics to tackle healthcare and drug discovery issues. Most recently, [8] discussed use of evolutionary algorithms to identify gender influence in rheumatic diseases.

Meta-heuristic algorithms include genetic algorithm (GA), differential evolution (DE) and particle swarm optimization (PSO), among many others. Each of these algorithms has been tested successfully in many real applications. The more popular ones like those just named have many variants, which improved the original versions in different ways. For example, the enhanced algorithms may converge faster, make the original algorithm less prone to premature convergence, or has a greater chance to extricate itself from a local optimum. An example of a swarm based meta-heuristic algorithm is Competitive Swarm Optimizer (CSO) and one of its variants is CSO-MA with mutated agents (CSO-MA). Hybridized algorithms that creatively combine suitable algorithms, meta-heuristic or not, can also markedly increase the performance of a meta-heuristic algorithm [9, 10]. A guiding principle is that the hybridized algorithm should perform better than either of the algorithms used in the hybridization.

The aim of this paper is to apply nature-inspired meta-heuristics to tackle a variety of challenging optimal design problems in the biosciences. We focus on one such algorithm proposed by one of the authors, called Competitive Swarm Optimizer with Mutating Agents (CSO-MA) and demonstrate its flexibility for solving a variety of more complex or high dimensional design problems. When it is possible to theoretically verify the optimality of a design, we also show how it can be verified using new innovative plots based on an equivalence theorem when the design space consists of all subsets of T positive integers. In the supporting material, we review CSO and CSO-MA and provide online Matlab and Python packages for running CSO-MA, along with web-based tools and references to monographs on meta-heuristics. We also include sme technical details for the applications.

In Section 2, we describe our statistical setup, approximate designs, optimality criteria and how to find and confirm optimality of an approximate design. The statistical models are described, including those for designing a longitudinal study. Section 3 reports a variety of optimal designs found from CSO-MA for different types of model and design criteria. They include locally D and c-optimal designs for different types of mixed nonlinear models, Bayesian optimal designs for estimating parameters in a HIV dynamics model and locally D-optimal designs for a high dimensional generalized linear models. Section 4 summarizes the key findings in the paper and emphasizes the broad applicability and usefulness of meta-heuristics for tackling hard-to-optimize problems, especially when all mathematical programming methods are not valid.

## 2 Statistical Models and Fundamentals of Optimal Design Construction

We first present statistical models and fundamentals in optimal design construction. Details can be found in design monographs, such as, [11] and [12].

Suppose the statistical model has the form E(y)=f(x,β), where y is the response, E(y) is the mean response that depends on a vector of explanatory factors x via a known nonlinear function f(x,β), apart from the unknown model parameters $\beta \in {\mathbb{R}}^{p}$. The responses may be uncorrelated or correlated and they need not necessarily be normally distributed. Given a design criterion, design issues concern how to optimally select values of the explanatory factors x from a pre-selected compact design space Ω to observe the response.

Designs can be exact or approximate. The main difference between the two types of designs is that the problem of finding an optimal approximate design is a convex optimization problem. Convex analysis theory then be used to construct algorithms for finding optimal approximate designs and confirming the optimality of an approximate design. In contrast, finding any optimal exact designs for a model requires solving a number-theoretical problem unless the regression model is extremely simple. There is no general theory for finding and confirming the optimality of an exact design. For this reason, we discuss mainly approximate designs but also provide examples of efficient exact optimal designs found by our algorithm.

Given an optimality criterion, approximate designs assume the given number of observations N is large and we determine the number n of design points in the optimal design, where these design points $x_{i}^{\prime }$ are and the proportions $w_{i}$ of observations to be taken at the design points. The design problem is to find optimal values of n and the pairs $\left(x_{i},w_{i}\right)$ subject to each proportion is positive, $w_{1}+\ldots +w_{n}=1$ and each $x_{i}\in \Omega$. Some approximate designs η with their weights and design points are displayed in Table 1.

The worth of a design η is measured its Fisher information matrix M(η,β) [13] for the model. When random effects are present, calculating the information matrix can be challenging unless the models are relatively simple. In practice, the matrix is often approximated before use. Design efficiency is mostly defined by the ratio of the design criterion values and that of the optimal design, with the exception of D efficiency defined by the $p^{th}$ root of the ratio of the two determinants. This is done to maintain the interpretation of of the design efficiency, scaled between 0 and 1 and then multiplied by 100. If a design has 50% efficiency, then the design has to be replicated twice for it to do as well as the optimum.

### 2.1 Optimality Criteria and Equivalence Theorems

In practice, design criteria are formulated as either concave or convex functions of the information matrix and common ones are D- and c-optimality. For nonlinear models, their information matrices depend on the unknown parameters which we want to estimate. To this end, we assume nominal parameter values of the parameters are available from either a pilot or similar [12, 14]. Because the optimal designs depend on the nominal values, they are only locally optimal. For instance, if $\beta^{0}$ is the vector of nominal values for β, the locally D-optimal design maximizes $log\left\{det\left(M\left(\eta ,\beta^{0}\right)\right)\right\}$ and the c-optimal design maximizes $-c^{T}M^{-1}\left(\eta ,\beta^{0}\right)c$ where c is an user-specified column vector if the aim is to estimate $c^{T}\;\beta$. In either case, the optimization is over all designs on the given compact design space.

The D and c-optimality criteria are both concave functions and so their directional derivatives always exist. We then use them to evaluate the D-optimality of a design η via a sensitivity function defined by

(1) $$S_{D}(\eta ,x)=\frac{\partial f{\left(x,\beta \right)}^{T}}{\partial \beta }M^{-1}(\eta ,\beta )\frac{\partial f(x,\beta )}{\partial \beta }-p,$$

where p is the number of parameters in the model. This leads to the general equivalence theorems discussed in [13] for checking optimality of a design among all design on the given design space Ω. For D-optimality, it asserts that the design $\eta^{⋆}$ is locally D-optimal if and only if $S_{D}\left(\eta^{⋆},x\right)\leq 0,\forall x\in \Omega$ with equality at the design points of $\eta^{⋆}$. Further details are available in [15], where equivalence theorems are derived for several convex (equivalently, concave) criteria. For example, the sensitivity function $S_{c}(\eta ,x)$ for local c-optimality of a design η is

(2) $$S_{c}(\eta ,x)={\left[\frac{\partial f{\left(x,\beta \right)}^{T}}{\partial \beta }M^{-1}(\eta ,\beta )c\right]}^{2}-c^{T}M^{-1}(\eta ,\beta )c$$

and $\eta^{⋆}$ is locally c-optimal if and only if $S_{c}\left(\eta^{⋆},x\right)\leq 0,\forall x\in \Omega$ with equality at all design points of $\eta^{⋆}$.

In what is to follow, we provide 7 applications of CSO-MA to demonstrate its flexibility and find various types of optimal designs for both linear and nonlinear with discrete or continuous outcome. Unlike the majority of work in this area, almost all models have random effects. Our designs can be exact and approximate designs and they can be locally or Bayesian optimal designs. The first two applications incorporate cost structure into the design construction and differs from some of the latest research in this area that often assumes a fixed effects model [16–18]. The last application concerns finding a Bayesian optimal design for a nonlinear model for studying HIV dynamics. There are recent packages/tools, such as that from [19], for finding optimal designs for nonlinear mixed models, but those are models specific and not amenable for finding some of the optimal designs reported herein.

In the implementation stage, we generally used ϕ=0.1 and a swarm size of 128 (except for application 3 and 6 we are using 256 and 16, respectively) in CSO-MA. If and when these inputs do not work as well, like other meta-heuristics, we change their values a bit. We assume nominal values are available for some of our applications; otherwise, we simulate and randomly choose a number of parameter sets for each model and then search for the optimal designs. The simulated nominal values for each model are shown, along with the CSO-MA generated design. All computations are done on a Windows 10 PC with a 3.20GHz Intel i7-8700 CPU, 32GB DDR4 2666MHz memory and 512G SSD storage. The programming language is MATLAB 2020a and Python 3.9.13. The codes are available from the first author.

## 3 Optimal Design Problems

In this section, we apply a state-of-art meta-heuristic algorithm to find a variety of new optimal designs under various complex situations. The rationale and descriptions of the selected meta-heuristic algorithms are given in the supporting material.

### 3.1 Experimental Costs

Costs are incurred when a measurement is made and may depend at which time point the observation is taken. Suppose the cost of taking an observation at time $\eta_{k}$ of the design η is given by a known function $c\left(\eta_{k}\right)$. We normalized the information matrix by the cost function and work with the normalized information matrix $M^{*}(\eta )$:

$$M^{\text{*}}(\eta )=\sum_{k=1}^{n}w_{k}\frac{M\left(\eta_{k}\right)}{c\left(\eta_{k}\right)}.$$

The advantages of incorporating cost into the design have been discussed in [20] and a common cost function is $c\left(\eta_{k}\right)=\alpha_{1}+\alpha_{2}n_{k}$, where $n_{k}$ is the number of time points/measurements taken at $\eta_{k}$, and $\alpha_{1},\alpha_{2}$ are user defined. The normalized information matrix is reminiscent of the common case seen in optimal design literature when errors are heteroscedastic and the inverse of the error variance at a point is represented by the efficiency function. When the cost structure is linear, the optimal design depends on the ratio of the two parameters $r=\alpha_{1}/\alpha_{2}$ and not on the values of $\alpha_{1}$ and $\alpha_{2}$. In the following subsections, we apply CSO-MA to generate designs for estimating parameters in various mixed models and some include cost considerations. All generated approximate designs have been verified to be optimal and for space consideration, we only show some of their sensitivity plots that confirm optimality of the CSO-MA generated designs.

### 3.2 Fractional Polynomial Regression Models

Suppose we wish to monitor the pulmonary function of the lungs of patients periodically in a clinical trial. A common measure is forced vital capacity, which is a continuous variable. Polynomials are traditionally used to model the outcome over time but increasingly, fractional polynomial models are used because they are more versatile and frequently provide better fits to the mean response over time [21]. Fractional polynomials are polynomials that allowed positive and negative fractions as powers for the monoials. [22] proposed such models and suggested that it is adequate to select powers of monoials from the set 𝒮={−2,−1,−0.5,0,0.5,1,2,3}. For our problem, we assume observations can be chosen from a discrete set $𝒯=\left\{1,2,\cdots ,T_{max}\right\}$, where $T_{max}$ is given and we want to determine the number and sampling time points for each subject in an optimal way.

Consider the linear fractional polynomial model with a subject random effect:

(3) $$y_{ij}=\beta_{0}+\beta_{1}t_{ij}^{-2}+\beta_{2}t_{ij}^{-1}+\beta_{3}t_{ij}^{-1/2}+\beta_{4}t_{ij}^{1/2}+\beta_{5}t_{ij}+\beta_{6}t_{ij}^{2}+\beta_{7}t_{ij}^{3}+b_{i}+e_{ij},$$

where we assume $b_{i}\;∼\;𝒩\left(0,\sigma_{b}^{2}\right),\;e_{ij}\;∼\;𝒩\left(0,\sigma_{e}^{2}\right)$. Here $t_{ij}$ refers to the time point of the j-th visit by individual $i,b_{i}$ is the random intercept term assumed to have a normal distribution with zero mean and variance $\sigma_{b}^{2},\;e_{ij}$ is an error term following a normal distribution with zero mean and variance $\sigma_{e}^{2}$. All random effects and error terms are assumed to be mutually independent of one another. Our interest is to find the locally D-optimal design to estimate the model parameters $\beta ={\left(\beta_{0},\cdots ,\beta_{7}\right)}^{T}$ by selecting the optimal subsets of time points from 𝒯 among all possible subsets from the pre-selected discretized design space.

Suppose η is a design with n design points $\eta_{1},\cdots ,\eta_{n}$ and each design point $\eta_{k}$ has $n_{k}$ time points $\left(t_{k1},\cdots ,t_{kn_{k}}\right)$. Let the weight for each subset of design points from 𝒯 for the design $\eta_{k}$ be $w_{j}$ subject to $\sum_{j=1}^{n}\;w_{j}=1$. Let $T_{k}$ be the $n_{k}\;\times \;8$ matrix whose l-th row is $\left\{1,t_{kl}^{-2},t_{kl}^{-1},t_{kl}^{-1/2},t_{kl}^{1/2},t_{kl},t_{kl}^{2},t_{kl}^{3}\right\}$, let $J_{n_{k}}$ be the $n_{k}\;\times \;n_{k}$ matrix whose elements are all equal to 1, let $I_{n_{k}}$ be the $n_{k}\times n_{k}$ identity matrix and let $SR=\sigma_{b}^{2}/\sigma_{e}^{2}$. A direct calculation shows

$$M\left(\eta_{k}\right)=T_{k}^{\prime }\Sigma_{k}^{-1}T_{k}=\frac{1}{\sigma_{e}^{2}}T_{k}^{\prime }{\left(I_{n_{k}}+\frac{\sigma_{b}^{2}}{\sigma_{e}^{2}}J_{n_{k}}\right)}^{-1}T_{k}\;=\frac{1}{\sigma_{e}^{2}}T_{k}^{\prime }\left(I_{n_{k}}-\frac{SR}{1+n_{k}SR}J_{n_{k}}\right)T_{k}.$$

**Application 1:** Suppose SR=2,r=0.5, and $T_{max}=10$. Table 1 shows that CSO-MA took 2.0 seconds of CPU time to find design $\eta_{1}$ with the D-criterion value of −33.200. This generated design has two groups of subjects, with about 83% of subjects having six measurements at times 1, 2, 3, 5, 8, 10 and 17% of subjects having six measurements at times 1, 2, 3, 6, 8, 10. This design guarantees that statistical inference for all the fixed parameters is estimated with maximum efficiency.

**Application 2:** Suppose SR=2,r=5, and $T_{max}=10$. Table 1 shows the CSO-MA generated design is $\eta_{2}$, which can be interpreted the same way as in the first case. It has a D-criterion value of −36.141 and CSO-MA took 2.0 seconds to find the design.

**Fig. 1:**
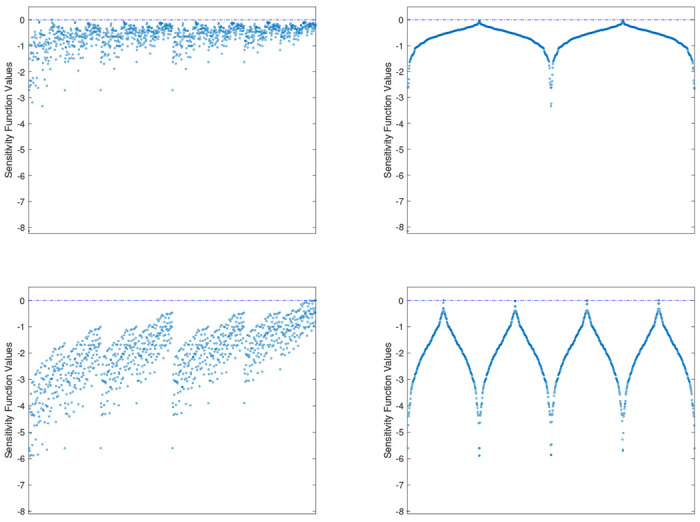
The sensitivity functions of the CSO-MA generated designs for Application 1 (first row) and Application 2 (second row) versus the design space comprising subsets of possible time points when they are appropriately ordered (right) and when they are not (left).

Figure 1 displays the sensitivity functions of the two CSO-MA generated designs for the two examples. Since $T_{max}=10$ hours and assuming the time interval is divided into {1,2,⋯,10}, there are $1023\left(2^{10}-1\right)$ possible sets of time points to observe a subject over the 10-hour period if the time unit is an hour, i.e., {1},{2},{3},⋯,{10},{1,2},{1,3},⋯,{1,2,⋯,10}. This means that the first choice requires the subjects be observed once at the end of the first 1 hour, and the last choice requires that the subject be observed every hour for 10 hours. The optimal approximate design selects what percentage of subjects be observed at different sets of time points and what the time points within each set. To construct the plot, we first order the sets of time points according to their values of the sensitivity function and then plot the function across the ordered 1023 sets of time points.

Figure 1 shows the uninformative plot of the sensitivity function on the left and the plot with properly ordered sets of time points on the right. The latter confirms the optimality of the generated designs for the two examples. We consider the right plot quite innovative since we have not seen one display under such a setting before.

**Application 3:** Fractional Polynomial Models with Correlated Random Coefficients We show that CSO-MA can generate optimal designs for models with multiple correlated random effects. Our example is illustrative in that the nominal model parameters and the covariance matrix of the random effects are arbitrarily selected. We assume we want to take four observations for each subject from the time interval t∈[1,10].

The model is

(4) $$y_{ij}=\beta_{0}+\beta_{1}t_{ij}^{-1/3}+\beta_{2}t_{ij}^{-1/2}+\beta_{3}t_{ij}^{1/3}+\beta_{4}t_{ij}^{1/2}+\beta_{5}t_{ij}+\beta_{6}t_{ij}^{2}+e_{ij},\;{\left(\beta_{0},\cdots ,\beta_{6}\right)}^{T}∼𝒩(0,D),\;e_{ij}∼𝒩\left(0,\sigma_{e}^{2}\right),$$

where

$$D=\text{Blockdiagonal}\left(D_{1},D_{2}\right),\text{with}\;D_{1}=\left(\begin{array}{ccc} 1.0 & 0.8 & 0.4\\ 0.8 & 1.2 & 0,5\\ 0.4 & 0.5 & 1.9 \end{array}\right),\;D_{2}=\left(\begin{array}{llll} 1.3 & 0.6 & 0.6 & 0.3\\ 0.6 & 1.2 & 0.7 & 0.4\\ 0.6 & 0.7 & 1.3 & 0.3\\ 0.3 & 0.4 & 0.3 & 1.0 \end{array}\right).$$

The information matrix is derived in a similar manner as in [23]. Since the value of $\sigma_{e}^{2}$ does not affect the optimization, we set it equal to 1. We used 256 particles and set ϕ=0.1 and the algorithm converged in 10~20 seconds.

Table 1 displays the locally CSO-MA generated design $\eta_{3}$ under the D-optimality criterion for Application 3. Its criterion value is −60.583. It shows that the total sample of subjects is divided unequally into four subgroups and patients in all the subgroup are observed at four time points, and these time points vary from subgroup to subgroup. For example, we observe from the first row of the design that one subgroup has 34.7% of the total number of subjects and each subject is observed at the time points 1.878, 4.224, 8.297 and 10.000. The other optimal designs are similarly interpreted.

### 3.3 Mixed Logistic Regression Models

Binary outcomes are ubiquitous and a logistic regression model with mixed-effects over time can be used to model the longitudinal data. The probability of a response from individual i at the time point $t_{j}$ may be described by a logistic model with a random intercept term and each time point may be chosen from a given discrete set $𝒯=\left\{1,2,\cdots ,T_{max}\right\}$.

We first consider a one-factor logistic mixed model. Suppose that the i-th subject has $n_{i}$ measurements at $t_{i1},\cdots ,t_{in_{i}}$ and $p_{ij}$ is the probability that the i-th subject responds to a treatment at time $t_{ij}$. Treating subject effect $b_{i}$ as random, we obtain

$$p_{ij}=\frac{\text{exp}\left(\beta_{0}+\beta_{1}t_{ij}+b_{i}\right)}{1+\text{exp}\left(\beta_{0}+\beta_{1}t_{ij}+b_{i}\right)},b_{i}∼𝒩\left(0,\sigma_{b}^{2}\right),\;i=1,\cdots ,n;\;j=1,\cdots ,n_{i}.$$

The research question is to find optimal time points for each patient to estimate the parameters $\beta_{0}$ and $\beta_{1}$ as accurately as possible.

The log-likelihood function of the above model does not have a closed form and the information matrix thus cannot be derived analytically. One solution is to use a first-order penalized quasi-likelihood (PQL) for approximating the true likelihood function [24,25]. By using the PQL method and assuming a n-point design η with each design point $\eta_{i}$ having $n_{i}$ time points/measurements, the information matrix can be approximated as

$$M\approx \sum_{i=1}^{n}T_{i}^{\prime }V_{i}^{-1}T_{i},\;V_{i}\;\approx \;W_{i}^{-1}+\sigma_{b}^{2}J_{n_{i}},$$

where $T_{i}$ is the i-th design matrix, J is a $n_{i}$-dimensional square matrix whose elements are all one’s and $W_{i}$ is a diagonal matrix given by

$$W_{i}=\mathrm{diag}\left[\mathrm{Var}\left(y_{i1}|b_{i}\right),\mathrm{Var}\left(y_{i2}|b_{i}\right),\cdots ,\mathrm{Var}\left(y_{in_{i}}|b_{i}\right)\right].$$

**Application 4:** A Logistic Model with Fractional Polynomials

The above model now has fractional polynomial terms in the link function given by

$$p_{ij}=\frac{\text{exp}\left(\beta_{0}+\beta_{1}t_{ij}^{-2}+\beta_{2}t_{ij}^{-1}+\beta_{3}t_{ij}^{-1/2}+\beta_{4}t_{ij}+b_{i}\right)}{1+\text{exp}\left(\beta_{0}+\beta_{1}t_{ij}^{-2}+\beta_{2}t_{ij}^{-1}+\beta_{3}t_{ij}^{-1/2}+\beta_{4}t_{ij}+b_{i}\right)},$$

where $b_{i}∼𝒩\left(0,\sigma_{b}^{2}\right)$. After the information matrix is approximated by the PQL method, CSO-MA searches for the optimal design. For example, we assume the values of the nominal parameters are: $\sigma_{b}^{2}=0.2,\beta =(1.0,0.2,-3.0,0.5,-1.2{)}^{T}$, the cost ratio is either r=6 or r=0.3 and we want to optimally choose time points $t_{ij}$ for each subject from the set {1,2,⋯,6}. Table 1 displays the locally CSO-MA generated D-optimal designs $\eta_{4-1}$ for r=6 and the D-optimal design $\eta_{4-2}$ for r=0.3. The CPU time for the first(second) search was 28(27) seconds and its criterion value is −49.381(−45.876).

These two optimal designs, along with those in Examples 1 and 2, suggest that more groups of subjects and more time points may be needed in the optimal design when the cost ratio in the linear cost function r becomes larger.

### 3.4 c-optimal Design for Negative Binomial Regression Models

To demonstrate the flexibility of our approach, we now design for a count model with mixed effects in a longitudinal study. The negative binomial regression model is a flexible model as it can be used to model over-dispersed or under-dispersed data. The count variable can be the number of new flares in Scleroderma patients or the number of new lesions in patients after a new treatment regimen over time. [26] found an optimal design for a phase I/II clinical trial for treating multiple sclerosis with gadoliniumenhanced lesions as the endpoint. The approach lacks theory and the design was selected among a few candidate designs and based entirely on simulated error rates produced by the various designs. Optimal designs for the Poisson and negative binomial regression models for fixed-effects or for models with a random intercept with one or two additive factors were reported in [27] and, [28], respectively.

Our models here are negative binomial mixed models and unlike previous work, we add a constraint that all subjects are observed at T user-selected number of points. Such a constraint arise in situations when, for example, taking observations are either laborious or expensive, or even risky in pediatric trials where only a limited number of measurements are allowed for the young subjects. In what is to follow, we show CSO-MA can directly accommodate such a constraint without difficulties. The derivations are modeled after [29] and are in the supporting information material for completeness.

As an application, we consider a two-drug trial and denote the combinations of the drug treatments by $x_{i}$ randomly assigned to the i-th subject and $x_{ik}$ is the dose level of drug k. We assume all drug levels have been normalized to [−1,1]. We further assume that once a drug level is determined for a patient, it remains unchanged throughout the trial. For administration purposes, each patient is required to observe T times given the space $\left\{1,2,3,\cdots ,T_{max}\right\}$ and $T_{max}$ is pre-determined. A negative binomial regression model is used to study the effects of the combinations of the drugs on the count outcome. The count outcome may be the number of allergy reactions after each treatment, or the number of new lesions seen after each treatment as in [26]. We chose the negative binomial regression model over the commonly-used Poisson regression model because the former is flexible and can capture under or over-dispersion.

Specifically, we have an additive negative binomial mixed model in two variables with a time trend and given by

(5) $$\text{log}\;\mu_{ij}=\beta_{0}+\beta_{1}x_{i1}+\beta_{2}x_{i2}+\theta t_{ij}+b_{i},\;b_{i}∼𝒩\left(0,\sigma_{b}^{2}\right),\;E\left(y_{ij}\right)=\mu_{ij},\;\mathrm{Var}\left(y_{ij}\right)=\mu_{ij}+a\mu_{ij},\;j=1,\cdots ,T.$$

Here the parameter a is the dispersion factor and if it is positive, it suggests that the data is over-dispersed, which is usually the case for real data. The information matrix of model (5) can also be approximated by the PQL method as in the last subsection.

**Application 5:** *c*-optimal Approximate Design for a Negative Binomial Model

Each subject in the clinical trial receives a combination dose from the two drugs and each subject is observed at only T=3 out of the $T_{max}=6$ possible time points with (one observation per week). The goals are to find an optimal design to determine what combination doses and which three-time points are best for answering the question: are the two drugs equally effective if the same dosage levels are given to the patients?

We find the locally c-optimal approximate designs with $c=(0,1,-1,0{)}^{T}$. For illustrative purposes, we assume the nominal model parameters are ${\left(\beta_{0},\beta_{1},\beta_{2}\right)}^{T}=(0.3,1.0,-0.5{)}^{T},\;\theta =1.1,\;a=1.2,\;\sigma_{b}^{2}=0.5,\;T_{max}=6$ and T=3. The ranges of values for the two independent variables are $x_{1}\in [-1,1],x_{2}\in [-1,1]$, after their dosage levels are appropriately scaled. Table 1 displays the generated design $\eta_{5}$ found by CSO-MA. Its criterion value is 0.501.

The CSO-MA generated design $\eta_{5}$ assigns equal number of patients to two dose combinations of the two drugs. Responses from each patient are observed three times, out of the 6 possible time points. The first group received a combination of the extreme doses from the two drugs and the second group received the other set of extreme doses from the two drugs. One group is observed at the first, second and sixth week, and the other group is observed at the second, fifth and sixth week.

### 3.5 Bayesian Optimal Exact Designs for Nonlinear Mixed Models Applied to HIV Dynamics

Finding Bayesian optimal designs for real applications is challenging because (i) the specifications of the nonlinear model and the prior distribution can be problematic, (ii) the design criterion does not have a closed form and consists of multiple integrals, which requires approximations using a random sampling scheme, and (iii) there is a lack of effective algorithms to numerically optimize the design criterion or an utility function under a general setting.

This subsection shows that CSO-MA can be effective to search for Bayesian optimal exact designs for estimating model parameters in a mixed-effects nonlinear model for a HIV trial studied by [30]. Recall that an exact design is characterized by a userspecified value of N, the total number of observations allowed for the study, and we want to determine the optimal number of sampling times, k, and the optimal number of replicates $n_{i}$ at the $i^{th}$ time point, $t_{i},i=1,\ldots ,k$ such that $n_{1}+\ldots +n_{k}=N$. Optimality is based on the user-specified design criterion.

Frequently, medical experts suggest a few designs that they believe are meaningful for the study. For instance, physicians believe that should be 6 sampling times in 2 hours and pharmacokinetic/pharmacodynamic (PK/PD) considerations may require more observations near the start and end of the sampling period. A few candidate designs are then proposed and for modelling purposes, a statistical model is postulated by the experts in PK/PD. If the goal is to estimate all model parameters in the model for prediction purposes, we compute the determinants of the information matrices for the few designs and implement the 6-point design with the largest determinant. The optimization is over all the pre-selected candidate designs and is not optimized among all 6-point designs. This suggests that even though there were a few pre-selected designs to choose from at the onset, additional options should be worth consideration if there are other designs that are noticeably more efficient and not too dissimilar from the candidate designs. We explore this possibility in this subsection.

**Application 6** There are 8 candidate designs for the HIV-dynamics model and each has 16 sampling time points from the time interval [0, 7]. The goal is to accurately estimate 3 parameters in the nonlinear mixed effects model and determine which one of the 8 designs is the optimum. The design criterion is to minimize the determinant of the covariance matrix of the 3 estimated parameters. The information matrix does not have a closed form and there are various ways to approximate the information matrix, see for example, [31]. The design criterion contains multiple integrals and the criterion has to be numerically optimized. The optimization can be among the 8 candidate designs with 16-points or among all 16-point designs on the interval [0, 7].

[30] used a Bayesian approach to determine which of the eight candidate designs is best. Two prior densities were used and the model used to study plasma concentration under the protease inhibitor monotherapy in the HIV dynamics study was a nonlinear mixed model. The information matrix for each design did not have a closed form and we approximated it using penalized quasi-likelihood methodology, as before. The information matirx of the design with the largest determinant value among the 8 candidate designs was then selected. In our work to follow, we use CSO-MA and find the best Bayesian exact optimal design among all designs with 16 time points.

Let $y_{ij}$ be the plasma concentration from subject i at time $t_{j}$ after the pharmacologic delay of the drug. Using the same notation and model in their paper, which is nonlinear mixed effects model, the natural logarithm of $y_{ij}$ is given by

$$y_{ij}|\theta_{i},t_{j},\sigma^{2}∼{𝒩}_{1}\left(s\left(\theta_{i},t_{j}\right),\sigma^{2}\right),\;j=1,\cdots ,T,$$

where ${𝒩}_{p}(u,V)$ is a p-dimensional normal distribution with mean u and covariance $V;\;\sigma^{2}$ is the noise level, $\theta_{i}$ is the random effect vector of subject $i,s\left(\theta_{i},t_{j}\right)$ is the nonlinear effect of subject i at time $t_{j}$. Further, we assume

(6) $$\theta_{i}={\left(\text{log}\;V_{0i},\text{log}\;c_{i},\;\text{log}\;\delta_{i}\right)}^{T}|\mu ,\Sigma ∼{𝒩}_{3}(\mu ,\Sigma ),\;s\left(\theta_{i},t_{j}\right)=\text{log}\;V_{0i}+\text{log}\;\left[\frac{c_{i}^{2}}{{\left(c_{i}-\delta_{i}\right)}^{2}}e^{-\delta_{i}t_{j}}-\frac{c_{i}^{2}-{\left(c_{i}\delta_{i}\right)}^{2}}{{\left(c_{i}-\delta_{i}\right)}^{2}}e^{-c_{i}t_{j}}-\frac{c_{i}\delta_{i}}{c_{i}-\delta_{i}}t_{j}e^{-c_{i}t_{j}}\right],$$

where $\mu ={\left(\mu_{1},\mu_{2},\mu_{3}\right)}^{T}\;=\;{\left(\log\;V_{0},\log\;c,\log\;\delta \right)}^{T}$ is the population level mean of $\theta_{i}$ and Σ is the population level covariance matrix. For subject $i,\;V_{0i}$ is the plasma concentration of HIV particles at treatment initiation; $c_{i}$ is the virion clearance rate and $\delta_{i}$ is the rate at which the infected CD4 cells die [32]. The expression of $s\left(\theta_{i},t_{j}\right)$ is obtained by solving a system of differential equations that describes the transactions among virus particles, target cells, and infected cells [33].

[32] proposed a more complicated hierarchical Bayesian model for studying HIV dynamics by allowing prior beliefs to be specified for the variance and covariance matrix as follows: The model is $y_{ij}|\theta_{i},t_{j},\sigma^{2}\;∼\;{𝒩}_{1}\left(s\left(\theta_{i},t_{j}\right),\sigma^{2}\right),\;j=1,\cdots ,T$, $\theta_{i}|\mu ,\Sigma ∼{𝒩}_{3}(\mu ,\Sigma ),\;i=1,\cdots ,n,\sigma^{-2}∼𝒢(\alpha ,\beta ),\mu ∼{𝒩}_{3}(\eta ,\Lambda )$ and $\Sigma^{-1}∼𝒲(\Phi ,\gamma )$, where 𝒢(α,β) is the Gamma distribution with shape α and rate β;𝒲(Φ,γ) is the Wishart distribution with scale matrix Φ and degrees of freedom γ. The $\theta_{i}$’s are random effects generated from ${𝒩}_{3}(\mu ,\Sigma )$. Other prior distributions may be used.

There are two particularly interesting parameters to estimate are $\mu_{2}=\log\;c$ and $\mu_{3}=\log\;\delta$. To incorporate prior information on the parameters, Bayesian design criteria were used to find time points $t={\left(t_{1},\cdots ,t_{T}\right)}^{T}$ that minimize the expectation of posterior variances $E\left[\mathrm{Var}\left(\mu_{2}|y\right)\right]$ and $E\left[\mathrm{Var}\left(\mu_{3}|y\right)\right]$, or equivalently, maximize the the utility functions, i.e., $-E\left[\mathrm{Var}\left(\mu_{2}|y\right)\right]$ and $-E\left[\mathrm{Var}\left(\mu_{3}|y\right)\right]$. [32] compared eight 16-point candidate designs and their performance is reported in Table 1 of [32].

Finding the Bayesian optimal design is not a trivial problem because we need to draw samples from the posterior distribution, which does not have an explicit form. The distribution of μ∣y in this application cannot be obtained analytically and we resort to the MCMC method to approximate the posterior distribution. Noting that

(7) $$f(\mu ,y)\;\propto \;\int \;f\left(y|\mu ,\Sigma^{-1},\sigma^{-2}\right)f(\mu )f\left(\Sigma^{-1}\right)f\left(\sigma^{-2}\right)d\Sigma^{-1}d\sigma^{-2},$$

where $f\left(y|\mu ,\Sigma^{-1},\sigma^{-2}\right)\;=\;\int \;f(y|\theta ,t)f\left(\theta |\mu ,\Sigma^{-1},\sigma^{-2}\right)d\theta$, we use the MetropolisHasting algorithm to draw random samples from f(μ∣y) and to estimate $E\left[\mathrm{Var}\left(\mu_{2}|y\right)\right]$ and $E\left[\mathrm{Var}\left(\mu_{3}|y\right)\right]$. Our algorithm for searching the Bayesian optimal design for this HIV dynamics model can be summarized as follows:

1. Given an input t and hyper parameters (α,β,η,Λ,Φ,γ), we hierarchically draw random samples for μ,Σ, and then θ and y.
2. Given an observation y, we use formula (7) and apply the M-H algorithm to estimate the posterior variance of μ∣y. A multivariate normal distribution is used as the proposal distribution, which is also the proposal distribution used in [32].
3. We apply CSO-MA to search the optimal design in the design space of t.

In Han’s paper, two prior distributions $\pi_{1},\pi_{2}$ were used with $\pi_{1}$ defined by $\sigma^{-2}\;∼\;𝒢(4.5,9.0),\;\mu \;∼\;{𝒩}_{3}\left(\left(11.0,1.1,-1.0{)}^{T},\mathrm{diag}(6.0,0.1,0.01\right)\right)$ and $\Sigma^{-1}\;∼\;𝒲(\mathrm{diag}(0.26,2.5,2.5),3.0)$. The prior $\pi_{2}$ is the same as $\pi_{1}$ except that the prior variance for $\mu_{3}$ is 0.001 instead of 0.01.

After we supplied the values of hyper-parameters of the prior distributions, we also have to supply values for M,N,K, whose definition can be found in the package file. The details of the whole procedure is given in the appendix.

Table 2 displays CSO-MA estimates for $E\left[\mathrm{Var}\left(\mu_{2}|y\right)\right]$ and $E\left[\mathrm{Var}\left(\mu_{3}|y\right)\right]$ and they agree with the results in Table 2 of [32]. This suggests hat the above three-step algorithm has worked well for this problem. For space considerations, we report CSO-MA results for candidate designs 1–4 only under the same two prior distributions in their paper. Table 3 displays the four CSO-MA generated 16-point Bayesian exact designs under each prior distribution and, as expected, all have smaller criterion values than all the candidate designs (see Table 2). The 4 percentages in parentheses in the last column of Table 3 are computed as follows using the first one 31.6% as an example. The smallest value of the numbers $E\left[\mathrm{Var}\left(\mu_{2}|y\right)\right]$ from the first 4 candidate designs under $\pi_{1}$ is 0.02882. Thus 100*(1−0.0197/0.02882)=31.6% is a conservative c-efficiency gain the Bayesian CSO-MA generated 16-point design has over the 4 candidate designs for estimating $E\left[\mathrm{Var}\left(\mu_{2}|y\right)\right]$. The other percentages in parentheses in the last column can be interpreted similarly. Noting that the c-efficiencies gain under the prior density $\pi_{2}$ are 26% and 71%, respectively, this suggests Bayesian exact optimal designs are sensitive to specification of the prior densities.

**Fig. 2:**
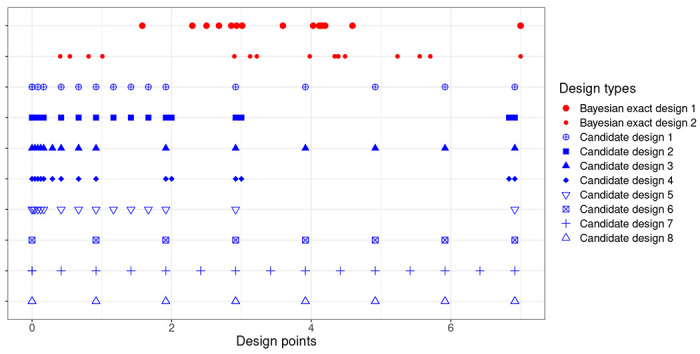
Design points from the Bayesian 16-point exact designs found by CSO-MA for estimating $E\left[\mathrm{Var}\left(\mu_{2}|y\right)\right]$ (first row) and for estimating $E\left[\mathrm{Var}\left(\mu_{3}|y\right)\right]$ (row 2). Both are determined under the prior density $\pi_{1}$. The next 8 rows display the design points of the 8 candidate designs in Han and Chaloner (2004).

Figure 2 displays the design points of the 8 candidate designs and those from the 16-point Bayesian designs found by CSO-MA for estimating $E\left[\mathrm{Var}\left(\mu_{2}|y\right)\right]$ and $E\left[\mathrm{Var}\left(\mu_{3}|y\right)\right]$ under prior density $\pi_{1}$. For space consideration, we omit results when the prior distribution is $\pi_{2}$ or corresponding results for estimating $\mu_{1}$. The figure shows that both the 16-point Bayesian optimal designs require one or more observations at the extreme point T=7 but no observation at the start of the study. They tend to be more concentrated in the interval [2,6] with that of estimating $\mu_{3}$ more spread out. Because the CSO-MA generated designs optimize the design criterion among all 16-point designs, we can calculate efficiencies of the candidate designs.

The message here is that with the CSO-MA generated designs, physicians can now be made aware of the statistical inefficiencies of the candidate designs and hopefully, the implemented design is an informed choice and represents a compromise between practical needs and statistical efficiency.

### 3.6 High Dimensional Locally D-optimal Design for Generalized Linear Models

In a generalized linear model (GLM) setting, there are usually multiple interacting factors (covariates) because a couple of explanatory factors may not capture the complex structure of the full data adequately. With more factors, there is an increasing number of parameters in the model. For example, a 5-factor GLM with all second order interactions has 16 parameters. Consequently, to find an optimal design to estimate all parameters, the design needs to have at least 16 points. This means we have to solve a constrained optimization problem of dimension 16×6=96 or more.

This subsection shows CSO-MA can find locally D-optimal designs for a high dimensional setting and that CSO-MA tends to outperform other meta-heuristics. Our models are the same to those in [34] so that we can compare CSO-MA results with their results obtained from particle swarm optimization (PSO), CSO and genetic algorithm (GA), which are well known meta-heuristic algorithms. Similar techniques can also be applied n to find locally optimal designs for higher dimensional models with more than 5 interacting factors. For example, [35] and [36], respectively, used differential evolution and quantum PSO, a variant of PSO, to find locally *D*-optimal designs for logistic models with 10 interacting factors.

**Application 7** We assume that we have logistic or Poisson regression models, all with 5 factors and include all two-factor interactions. There are different sets of nominal values and the goal is to find a locally *D*-optimal design for estimating all parameters in the model. Following [37], the Fisher information matrix of a design ξ with n observations and nominal value θ for the parameters is given by

$$M(\xi ,\theta )=\sum_{i=1}^{n}p_{i}x_{i}x_{i}^{T},$$

where $x_{i}={\left(1,x_{i1},\cdots ,x_{i5},x_{i1}x_{i2},\cdots ,x_{i4}x_{i5}\right)}^{T}$ is the $i^{th}$ row of the design matrix. Here $p_{i}$ is the variance of the $i^{th}$ observation at $x_{i},i=1,2,\cdots$, For Poisson models with mean parameter $\lambda_{i},p_{i}=\lambda_{i}$ and for logistic models with mean parameter $\lambda_{i}$, $p_{i}=\lambda_{i}\left(1-\lambda_{i}\right)$.

The goal is to find a locally D-optimal approximate design $\xi^{*}$ for estimating all parameters in the mean function. This means that we have to determine the optimal number k of support points in the design, the support points $x_{i}$ and the proportion $\omega_{i}$ of the n observations to be observed at $x_{i},\;i=1,\ldots ,k$, i.e.

$$\xi^{\text{*}}=\text{arg}\;\underset{\xi }{\text{max}}\;\text{logdet}\left(\sum_{i=1}^{k}w_{i}p_{i}x_{i}x_{i}^{T}\right).$$

We note that the number of design points k cannot be less than the number of parameters (which is 16 in this case); otherwise the information matrix is singular. Our experience is that it is helpful to start algorithm and search for the optimum using a large value for k to initiate the algorithm. This is because the algorithm can only find a design with up to k support point only. A rough guide for the choice for k is to choose its value about twice the number of parameters in the model.

Table 4 displays the simulated criterion values of designs found by CSO-MA for the two logistic models and the two Poisson models, along with their standard deviations and average run times. All models have different nominal parameter values and they are shown in Table 1 in [34]. Compared with results Table 2 of [34], it is evident that CSO-MA clearly and consistently outperforms the 3 other meta-heuristic algorithms: GA, PSO and CSO. The outperformance of CSO-MA relative to CSO is not surprisingly because the former is a variant of CSO. The overall comparison results show that CSO-MA is able to produce more stable and more efficient designs with shorter run time compared with other meta-heuristic algorithms. As an example, Table 5 shows the locally D-optimal design found by CSO-MA for Poisson Model 2. This PSO-generated design has 16 design points, 5 fewer than that found in [34] and higher efficiency. Having fewer points in a design can be attractive if incurring observations at different time points is costly or laborious.

## 4 Discussion

Finding optimal designs for a nonlinear mixed-effects model with possibly correlated random effects and several interacting factors is challenging and under-researched. This paper demonstrates the utility of a nature-inspired meta-heuristic algorithm in tackling complex design problems for different types of models. The models can be linear or nonlinear, have random effects or not and errors can be correlated or not. As demonstrated in the first two applications, they can also incorporate cost structure in the design construction. The method also works for design problems with a prefixed number of possible time points and we want to find an approximate design from the design space that now consists of all possible subsets from the set {1,2,…,T} and T is the predetermined end time for the study. Using CSO-MA, we found an optimal design among all designs defined on such a design space and provide innovative plots to confirm its optimality. We focus on D and c-optimality, but the metah-heuristic algorithm CSO-MA should also be able to find optimal designs under other criteria, including Bayesian and optimal exact designs. We also showed that the proposed algorithm either outperforms or performs as well as its competitors in several setups.

We close by noting that nature-inspired meta-heuristic algorithms are generalpurpose optimization tools and their applications are not limited to solving design problems, or even optimization problems. For example, they can also be used creatively to solve a system of nonlinear equations [38]. In summary, meta-heuristic algorithms, and particularly nature-inspired meta-heuristic algorithms, are especially useful when conventional algorithms or mathematical programming approaches are not applicable or fail to provide a quality solution to an optimization problem.

## Figures and Tables

**Table 1:** The CSO-MA generated designs for the 5 examples.

Application 1	Application 2
$\eta_{1}=\left(\begin{array}{c} \left\{1,2,3,5,8,10\right\}0.832\\ \left\{1,2,3,6,8,10\right\}0.168 \end{array}\right)$	$\eta_{2}=\left(\begin{array}{c} \left\{1,2,4,6,8,9,10\right\}0.100\\ \left\{1,2,3,5,6,8,9,10\right\}0.260\\ \left\{1,2,3,4,6,8,10\right\}0.275\\ \left\{1,2,3,5,6,8,9,10\right\}0.365 \end{array}\right)$

Application 3	Application 4-1
$\eta_{3}=\left(\begin{array}{c} \left\{1.878,4.224,8.297,10.000\right\}0.347\\ \left\{1.000,1.254,2.015,3.910\right\}0.311\\ \left\{1.282,3.000,6.070,9.053\right\}0.189\\ \left\{1.000,3.116,6.132,9.114\right\}0.153 \end{array}\right)$	$\eta_{4-1}=\left(\begin{array}{c} \left\{1,2,3,4,6\right\}0.500\\ \left\{1,2,3,4,5,6\right\}0.500 \end{array}\right)$

Application 4-2	Application 5
$\eta_{4-2}=\left(\left\{1,2,3,4,6\right\}1.000\right)$	$\eta_{5}=\left(\begin{array}{cccccc} x_{1} & x_{2} & t_{1} & t_{2} & t_{3} & w\\ -1.000 & 1.000 & 2 & 5 & 6 & 0.500\\ 1.000 & -1.000 & 1 & 2 & 6 & 0.500 \end{array}\right)$

**Table 2:** The estimated values of $E\left[\mathrm{Var}\left(\mu_{2}|y\right)\right]$ and $E\left[\mathrm{Var}\left(\mu_{3}|y\right)\right]$ for the candidate designs 1-4 produced from our implemented 3-step algorithm. The corresponding standard deviation (std) of the criterion value averaged over 10 repetitions is also reported. The results from our implemented 3-step algorithm are very close to the results in Han and Chaloner (2004).

	$\pi_{1}$		$\pi_{2}$	
	$\overset{ˆ}{E}\left[\mathrm{Var}\left(\mu_{2}|y\right)\right]$	Std	$\overset{ˆ}{E}\left[\mathrm{Var}\left(\mu_{2}|y\right)\right]$	Std

Candidate design 1	0.02915	0.0029	0.02968	0.0014
Candidate design 2	0.02882	0.0017	0.02874	0.0019
Candidate design 3	0.03257	0.0031	0.03315	0.0023
Candidate design 4	0.03003	0.0026	0.02946	0.0018
	$\overset{ˆ}{E}\left[\mathrm{Var}\left(\mu_{3}|y\right)\right]$	Std	$\overset{ˆ}{E}\left[\mathrm{Var}\left(\mu_{3}|y\right)\right]$	Std

Candidate design 1	0.005172	0.00030	0.01117	0.0014
Candidate design 2	0.005298	0.00014	0.01120	0.0019
Candidate design 3	0.005367	0.00012	0.01103	0.0023
Candidate design 4	0.005330	0.00009	0.01089	0.0011

**Table 3:** Bayesian optimal designs found by CSO-MA for minimizing $E\left[\mathrm{Var}\left(\mu_{2}|y\right)\right]$ and $E\left[\mathrm{Var}\left(\mu_{3}|y\right)\right]$ under prior $\pi_{1}$ and $\pi_{2}$, along with their simulated criterion values in the last column; the percentage in brackets shows the relative decrease in criterion values compared with the minimum values reported in Table 2.

Objective	Prior	Design								Criterion value
$E\left[\mathrm{Var}\left(\mu_{2}|y\right)\right]$	$\pi_{1}$	1.580	2.297	2.500	2.678	2.855	2.932	3.011	3.594	0.0197 (31.6%)
		4.028	4.115	4.146	4.155	4.200	4.591	7.000	7.000	

$E\left[\mathrm{Var}\left(\mu_{2}|y\right)\right]$	$\pi_{2}$	0.000	0.017	0.473	1.224	1.490	1.901	1.997	2.400	0.0214 (25.5%)
		3.278	4.109	5.280	5.635	5.761	6.363	7.000	7.000	

$E\left[\mathrm{Var}\left(\mu_{3}|y\right)\right]$	$\pi_{1}$	0.404	0.542	0.810	1.008	2.899	3.125	3.220	3.979	0.0023 (55.5%)
		4.336	4.379	4.389	4.485	5.236	5.554	5.706	7.000	

$E\left[\mathrm{Var}\left(\mu_{3}|y\right)\right]$	$\pi_{2}$	0.000	0.212	1.078	1.588	1.792	2.711	2.901	2.933	0.0026 (76.1%)
		3.072	3.583	3.600	3.818	3.950	4.062	4.561	6.575	

**Table 4:** Average simulated criterion values of the locally D-optimal designs found by different algorithms, along with their standard deviations in parentheses. The simulated results suggest that CSO-MA outperforms the other 3 algorithms in terms of criterion values, standard errors and average runtime.

Model	CSO-MA	GA	PSO	CSO
Logistic 1	−28.88(0.16)	−29.54(0.83)	−31.05(1.51)	−28.80(0.37)
Logistic 2	−28.89(0.22)	−29.76(1.12)	−30.78(1.07)	−28.91(0.54)
Poisson 1	151.67(0.41)	167.30(1.31)	163.11(0.92)	169.04(1.24)
Poisson 2	100.51(0.32)	100.14(1.71)	93.23(1.40)	100.35(0.64)
Average Runtime	20.1s	95.2s	64.5s	64.5s

**Table 5:** A CSO-MA generated 16-point design for Poisson model 2. Each row represents a design point with the last column showing the weight of the design at each point.

*x*_1_	*x*_2_	*x*_3_	*x*_4_	*x*_5_	w
1.000	−1.000	0.001	1.000	−1.000	0.0629
−1.000	−1.000	−1.000	1.000	−0.505	0.0628
−1.000	1.000	−0.683	−1.000	−1.000	0.0624
−1.000	−0.452	−1.000	1.000	−0.523	0.0625
−1.000	1.000	−1.000	−1.000	−1.000	0.0627
−0.030	−1.000	−1.000	1.000	−0.600	0.0626
−1.000	−0.436	−1.000	1.000	−1.000	0.0627
0.906	−1.000	−1.000	0.222	−1.000	0.0622
−1.000	−1.000	−1.000	1.000	−1.000	0.0622
0.195	−1.000	−1.000	1.000	−1.000	0.0617
−1.000	−1.000	−0.670	1.000	−1.000	0.0627
−1.000	1.000	−1.000	−1.000	0.506	0.0636
−1.000	−0.350	−0.618	1.000	−1.000	0.0627
−1.000	−1.000	−0.611	1.000	−0.419	0.0623
−0.198	1.000	−1.000	−1.000	−1.000	0.0618
−1.000	−1.000	−1.000	0.516	−1.000	0.0622

## Appendix A Nature-Inspired Meta-heuristic Algorithms

Nature-inspired meta-heuristic algorithms, such as swarm-based algorithms, now form a dominant component in the optimization literature. [39, 40] documented their meteoric rise in popularity in solving real, high-dimensional and complex optimization problems, and their interest and growth has continued unabated. They are already widely used in engineering and computer science, and recently also in other disciplines for tackling high-dimensional and complex real optimization problems. There are many reviews of such algorithms and their many multi-faceted applications to solve very different types of challenging optimization problems; see, for example, [41], and [42], among several others. They have several appealing features over current algorithms and the main ones are that their codes are widely and freely available, they are easy to implement and use; they are fast, essentially assumptions free and are general-purpose optimization algorithms. While they do not guarantee that they will find the optimal solution, they frequently do so or find a nearly optimal solution quickly. Some recent studies have shown that swarm-based algorithms can search for hard-to-find optimal designs that were previously deemed difficult. For instance, [43] found optimal designs for standardized maximin optimal designs for nonlinear models and [35] found optimal designs for logistic models with many interacting factors.

There are many monographs on nature-inspired meta-heuristic algorithms at various levels; for instance, [44] monograph is at an introductory level and [45] is at a bit more advanced level. Some monographs target specific disciplines; for example, [46] focus on agricultural applications and [47] is interested in feature selection problems and has numerous applications in finance and reinforce learning. metaheuristics has undoubtedly emerged as a set of dominant optimization tools in industry and academia [39] and in artificial intelligence research as well [1]. Their applications are plentiful and have been shown capable of solving different types of complex and high-dimensional optimization problems [48–50]. Websites like R, Python and MATLAB codes are available at youtube.com, github.com, including at professional websites, such as Matlab.com and SAS.com. For example, the website https://pyswarms.readthedocs.io/en/latest/ has a set of PSO tools written in Python [51]. See also https://github.com/ElvisCuiHan/CSOMA, where codes are available for using CSO-MA to solve different types of estimation and design problems in statistics, including imputing missing data optimally or in the optimal selection of variables in ecology. [52] provided a high-level Python package for selecting machine learning algorithms and their parameters using PSO.

### A.1 Competitive Swarm Optimizer

Competitive Swarm Optimizer (CSO) is a relatively novel swarm-based algorithm that has been proven to be very effective in solving different types of optimization problems [53]. CSO has had successful applications to solve large and hard optimization problems. For example, [54] applied CSO to select variables for high-dimensional classification models, and [55] used CSO to study a power system economic dispatch, which is typically a complex nonlinear multivariable strongly coupled optimization problem with equality and inequality constraints.

Competitive Swarm Optimizer algorithm, or CSO for short, minimizes a given function f(x) over a user-specified compact space Ω by first generating a set of candidate solutions. In our case, they take the form of a swarm of n particles at positions $x_{1},\cdots ,x_{n}$, along with their corresponding random velocities $v_{1},\cdots ,v_{n}$.

After the initial swarm is generated, at each iteration we randomly divide the swarm into $\left\lfloor \frac{n}{2}\right\rfloor$ pairs and compare their objective function values. We identify $x_{i}^{t}$ as the winner and $x_{j}^{t}$ as the loser if these two are competed at the iteration t and $f\left(x_{i}^{t}\right)\;<\;f\left(x_{j}^{t}\right)$. The winner retains the status quo and the loser learns from the winner. The two defining equations for CSO are

(A1)
$$v_{j}^{t+1}=R_{1}⊗v_{j}^{t}+R_{2}⊗\left(x_{i}^{t}-x_{j}^{t}\right)+\phi R_{3}⊗\left({\bar{x}}^{t}-x_{j}^{t}\right)$$

(A2)
$$\text{and}\;x_{j}^{t+1}=x_{j}^{t}+v_{j}^{t+1},$$

where $R_{1},R_{2},R_{3}$ are all random vectors whose elements are drawn from U(0,1); operation ⊗ also represents element-wise multiplication; vector ${\bar{x}}^{t}$ is simply the swarm center at iteration t; social factor ϕ controls the influence of the neighboring particles to the loser and a large value is helpful for enhancing swarm diversity (but possibly impacts convergence rate). This process iterates until some stopping criteria are met.

Simulation results have repeatedly shown that CSO either outperforms or is competitive with several state-of-the-art evolutionary algorithms, including several enhanced versions of PSO. This conclusion was arrived at after comparing CSO performance with state-of-the-art EAs using a variety of benchmark functions with dimensions up to 5000 and found that CSO was frequently the fastest and winner in terms of the quality of the solution [53, 56–59].

### A.2 Competitive Swarm Optimizer with Mutated Agents

Our experience with CSO is that many of the generated design points are at the boundary of the design space Ω. This is helpful because many optimal designs, especially *D*-optimal designs, have their support points at the boundary Ω.

To this end, we propose an improvement on CSO and call the enhanced version, Competitive Swarm Optimizer with Mutated Agents or, in short, CSO-MA. After pairing up the swarm in groups of two at each iteration, we randomly choose a loser particle p as an agent, randomly pick a variable indexed as q and then randomly change the value of $x_{pq}$ to either ${xmax}_{q}$ or ${xmin}_{q}$, where ${xmax}_{q}$ and ${xmin}_{q}$ represent, respectively, the upper bound and lower bound of the q-th variable. If the current optimal value is already close to the global optimum, this change will not hurt since we implement this experiment on a loser particle, which is not leading the movement for the whole swarm; otherwise, this chosen agent restarts a journey from the boundary and has a chance to escape from a local optimum.

The computational complexity of CSO is 𝒪(nD), where n is the swarm size and D is the dimension of the problem. Since our modification only adds one coordinate mutation operation to each particle, its computational complexity is the same as that of CSO. The improved performance of CSO-MA over CSO-MA to find the optimum for many complex multi-dimensional benchmark functions has been validated [60].

## Appendix B Derivation of the Information Matrix

Suppose we have a negative binomial model with a random intercept and a single fixed factor. Suppose further that there are n subjects, each with T independent observations and $\mu_{ij}$ is the mean of the count outcome $y_{ij}$. Then

$$\text{log}\;\mu_{ij}=\beta_{0}+\beta_{1}x_{i1}+b_{i},\;b_{i}∼𝒩\left(0,\sigma_{b}^{2}\right),\;i=1,\cdots ,n,j=1,\cdots ,T.$$

[29] showed that the total likelihood function for such a mixed model is

$$L=\prod_{i=1}^{n}\prod_{j=1}^{T}\frac{\text{Γ}\left(y_{ij}+\frac{1}{a}\right)}{\text{Γ}\left(\frac{1}{a}\right)}{\left(\frac{a\mu_{ij}}{1+a\mu_{ij}}\right)}^{y_{ij}}{\left(\frac{1}{1+a\mu_{ij}}\right)}^{\frac{1}{a}},$$

where a is the dispersion factor. Apart from an unimportant additive constant, the log-likelihood is

$$l=\text{log}\;L=\sum_{i=1}^{n}\sum_{j=1}^{T}y_{ij}\text{log}\frac{a\mu_{ij}}{1+a\mu_{ij}}-\sum_{i=1}^{n}\sum_{j=1}^{T}\frac{1}{a}\text{log}\left(1+a\mu_{ij}\right).$$

Noting that

$$\mu_{ij}=\int e^{\beta_{0}+\beta_{1}x_{i1}+b_{i}}\frac{1}{\sqrt{2\pi }\sigma_{b}}e^{-\frac{b_{i}^{2}}{2\sigma_{b}^{2}}}db_{i}=e^{\beta_{0}+\beta_{1}x_{i1}+\frac{\sigma_{b}^{2}}{2}}\int \frac{1}{\sqrt{2\pi }\sigma_{b}}e^{-\frac{{\left(b_{i}-\sigma_{b}^{2}\right)}^{2}}{2\sigma_{b}^{2}}}db_{i}=e^{\beta_{0}+\beta_{1}x_{i1}+\frac{\sigma_{b}^{2}}{2}}.$$

we calculate the following quantities for the Fisher information matrix:

$$\frac{\partial l}{\partial \beta_{0}}=\sum_{i=1}^{n}\sum_{j=1}^{T}y_{ij}\frac{1}{1+ae^{\beta_{0}+\beta_{1}x_{i1}+\frac{\sigma_{b}^{2}}{2}}}-\sum_{i=1}^{n}\sum_{j=1}^{T}\frac{e^{\beta_{0}+\beta_{1}x_{i1}+\frac{\sigma_{b}^{2}}{2}}}{1+ae^{\beta_{0}+\beta_{1}x_{i1}+\frac{\sigma_{b}^{2}}{2}}},\;\frac{\partial l}{\partial \beta_{1}}=\sum_{i=1}^{n}\sum_{j=1}^{T}y_{ij}\frac{x_{i1}}{1+ae^{\beta_{0}+\beta_{1}x_{i1}+\frac{\sigma_{b}^{2}}{2}}}-\sum_{i=1}^{n}\sum_{j=1}^{T}\frac{x_{i1}e^{\beta_{0}+\beta_{1}x_{i1}+\frac{\sigma_{b}^{2}}{2}}}{1+ae^{\beta_{0}+\beta_{1}x_{i1}+\frac{\sigma_{b}^{2}}{2}}},\;\frac{\partial^{2}l}{\partial \beta_{0}^{2}}=-\sum_{i=1}^{n}\sum_{j=1}^{T}\frac{e^{\beta_{0}+\beta_{1}x_{i1}+\frac{\sigma_{b}^{2}}{2}}}{{\left(1+ae^{\beta_{0}+\beta_{1}x_{i1}+\frac{\sigma_{b}^{2}}{2}}\right)}^{2}}\left(ay_{ij}+1\right),\frac{\partial^{2}l}{\partial \beta_{0}\beta_{1}}=-\sum_{i=1}^{n}\sum_{j=1}^{T}\frac{x_{i1}e^{\beta_{0}+\beta_{1}x_{i1}+\frac{\sigma_{b}^{2}}{2}}}{{\left(1+ae^{\beta_{0}+\beta_{1}x_{i1}+\frac{\sigma_{b}^{2}}{2}}\right)}^{2}}\left(ay_{ij}+1\right),\;\frac{\partial^{2}l}{\partial \beta_{1}^{2}}=-\sum_{i=1}^{n}\sum_{j=1}^{T}\frac{x_{i1}^{2}e^{\beta_{0}+\beta_{1}x_{i1}+\frac{\sigma_{b}^{2}}{2}}}{{\left(1+ae^{\beta_{0}+\beta_{1}x_{i1}+\frac{\sigma_{b}^{2}}{2}}\right)}^{2}}\left(ay_{ij}+1\right),E_{y_{ij}}\left(-\frac{\partial^{2}l}{\partial \beta_{0}^{2}}\right)=\sum_{i=1}^{n}\sum_{j=1}^{T}\frac{e^{\beta_{0}+\beta_{1}x_{i1}+\frac{1}{2}}}{1+ae^{\beta_{0}+\beta_{1}x_{i1}+\frac{\sigma_{b}^{2}}{2}}},\;E_{y_{ij}}\left(-\frac{\partial^{2}l}{\partial \beta_{0}\beta_{1}}\right)=\sum_{i=1}^{n}\sum_{j=1}^{T}\frac{x_{i1}e^{\beta_{0}+\beta_{1}x_{i1}+\frac{\sigma_{b}^{2}}{2}}}{1+ae^{\beta_{0}+\beta_{1}x_{i1}+\frac{\sigma_{b}^{2}}{2}}},E_{y_{ij}}\left(-\frac{\partial^{2}l}{\partial \beta_{1}^{2}}\right)=\overset{n}{\underset{i=1}{\sum }}\sum_{j=1}^{T}\frac{x_{i1}^{2}e^{\beta_{0}+\beta_{1}x_{i1}+\frac{1}{2}}}{1+ae^{\beta_{0}+\beta_{1}x_{i1}+\frac{\sigma_{b}^{2}}{2}}}.$$

The information matrix for a mixed negative binomial model with a random intercept and multiple fixed factors can be derived similarly.

## Appendix C Details on the Bayesian Optimal Design for Nonlinear Mixed Models Applied to HIV Dynamics

In this subsection, we give details on computing the utility function $E\left[\mathrm{Var}\left(\mu_{2}|y\right)\right]$ for the HIV dynamics model. Details for computing $E\left[\mathrm{Var}\left(\mu_{3}|y\right)\right]$ are similar and therefore omitted. Mathematically, the utility function can be written as

$$E\left[\mathrm{Var}\left(\mu_{2}|y\right)\right]=E\left(\mu_{2}^{2}\right)-E\left[E^{2}\left(\mu_{2}|y\right)\right].$$

Recall that μ∼𝒩(η,Λ), so the first term does not involve $t_{j}$ because the second moment of $\mu_{2}$ is just $\eta_{2}^{2}+\Lambda_{22}$ where $\eta_{2}$ is the second element of η and $\Lambda_{22}$ is the $(2,2{)}^{th}$ element of Λ. Hence optimization with respect to $t_{j}$ only involves the second term and according to theorem 1 in [32], it is finite. It requires the computation of

$$E\left(\mu_{2}|y\right)=\int \;\mu_{2}f(\mu |y)d\mu \;\propto \;\int \;\mu_{2}f(\mu ,y)d\mu \;=\int \;\mu_{2}f\left(y|\mu ,\Sigma^{-1},\sigma^{-2}\right)f\left(\mu ,\Sigma^{-1},\sigma^{-2}\right)d\mu d\Sigma^{-1}d\sigma^{-2}.$$

Recall that for fixed i and j, we have $f\left(y_{ij}|\mu ,\Sigma^{-1},\sigma^{-2}\right)=\int \;f\left(y_{ij}|\theta_{i},t_{j},\sigma^{-2}\right)f\left(\theta_{i}|\mu ,\Sigma \right)d\theta_{i}$, where $\theta_{i}={\left(\log V_{0i},\log\;c_{i},\log\;\delta_{i}\right)}^{T}$ is defined in equation (6) in the paper. The density $f\left(y_{i}|\mu ,\Sigma^{-1},\sigma^{-2}\right)$ can be approximated as follows (for a fixed design $t_{ij},j=1,\cdots ,T$):

(C3)
$$f\left(y_{i}|\mu ,\Sigma^{-1},\sigma^{-2}\right)=\int \;\prod_{j=1}^{T}f\left(y_{ij}|\theta_{i},t_{j},\sigma^{-2}\right)f\left(\theta_{i}|\mu ,\Sigma^{-1}\right)d\theta_{i}\;\approx \frac{1}{K}\sum_{k=1}^{K}\prod_{j=1}^{T}f\left(y_{ij}|\theta_{k},t_{j},\sigma^{-2}\right)f\left(\theta_{k}|\mu ,\Sigma^{-1}\right)\;=\sum_{k=1}^{K}\frac{1}{{\left(2\pi \right)}^{2}\sigma \sqrt{\left|\Sigma \right|}K}\prod_{j=1}^{T}\text{exp}\left\{-\frac{{\left[y_{ij}-s\left(\theta_{k},t_{j}\right)\right]}^{2}}{2\sigma^{2}}-\frac{1}{2}\left(\theta_{k}-\mu \right)\Sigma^{-1}{\left(\theta_{k}-\mu \right)}^{\prime }\right\},$$

where T is the number of time points (T=16 in our paper) and K is the number of quadratures. We found that K=5 is sufficient to obtain stable results. We denote the above approximation by $F_{1}\left(t,y_{i},\mu ,\Sigma^{-1},\sigma^{-2},K\right)$. Next, we use a sampling scheme to derive i.i.d. samples $\left(\mu_{i},\Sigma_{i}^{-1},\sigma_{i}^{-2}\right)$ for i=1,⋯,s, and hence

(C4)
$$E\left(\mu_{2}|y\right)\;\propto \;\frac{1}{N}\sum_{i=1}^{N}F_{1}\left(t,y_{i},\mu_{i},\Sigma_{i}^{-1},\sigma_{i}^{-2},K\right)f\left(\mu_{i},\Sigma_{i}^{-1},\sigma_{i}^{-2}\right)\mu_{i2},$$

where N=5 gives stable results and

$$f\left(\mu ,\Sigma^{-1},\sigma^{-2}\right)={𝒩}_{3}(\mu |\eta ,\Lambda )\times 𝒲\left(\Sigma^{-1}|\Phi ,\gamma \right)\times 𝒢\left(\sigma^{-2}|\alpha ,\beta \right)\;=\frac{1}{{\sqrt{2\pi }}^{3}\sqrt{\left|\Lambda \right|}}\text{exp}\left[-\frac{1}{2}(\mu -\eta )\Lambda^{-1}{\left(\mu -\eta \right)}^{\prime }\right]\times \;\frac{{\left|\Sigma^{-1}\right|}^{\frac{\gamma -3-1}{2}}\text{exp}\left[-\mathrm{tr}\left(\Phi^{-1}\Sigma^{-1}\right)/2\right]}{2^{\frac{3\gamma }{2}}|\Phi {|}^{\frac{\gamma }{2}}{\text{Γ}}_{3}\left(\frac{\gamma }{2}\right)}\frac{\beta^{\alpha }}{\text{Γ}(\alpha )}{\left(\sigma^{-2}\right)}^{\alpha -1}\text{exp}\left(-\beta \sigma^{-2}\right),$$

and

$${\text{Γ}}_{3}\left(\frac{\gamma }{2}\right)=\pi^{1.5}\text{Γ}\left(\frac{\gamma }{2}\right)\text{Γ}\left(\frac{\gamma }{2}-0.5\right)\text{Γ}\left(\frac{\gamma }{2}-1\right).$$

We denote the above approximation (C4) as $F_{2}\left(t,y_{i},K,\Phi ,\gamma ,\eta ,\Lambda ,\alpha ,\beta ,N\right)$. We then draw S different y samples to approximate the outer expectation:

(C5)
$$E\left[E^{2}\left(\mu_{2}|y\right)\right]\approx \frac{1}{S}\sum_{i=1}^{S}F_{2}^{2}\left(t,y_{i},K,\Phi ,\gamma ,\eta ,\Lambda ,\alpha ,\beta ,N\right)$$

To sum up, we use the following algorithm to generate a Bayesian design:

- We start with a sequence of random y, say, generated uniformly from −10 to 10.
- Given the initial y, we apply CSO-MA to find the optimal t.
- Given the optimal t we obtain in Step 2, we resample y.
- Iterate between Steps 2 and 3 until some user-specified stopping criterion is met. In our case, we run 30 times in total and we set T=16,N=5,K=5 and S=50.

## Appendix D On the Online CSOMA Package

The CSOMA algorithm was originally written in Matlab and is available online at https://github.com/ElvisCuiHan/CSOMA/tree/main/CSOMA%for%20Matlab. We extend it to Python, utilizing the *pyswarms* package [51] and it is available at https://github.com/ElvisCuiHan/CSOMA/tree/main/CSOMA%for%20Python. On the same page, an example for finding D-optimal design in a trinomial dose-response model is given.

In addition, codes for application 3 and 7 are also given in https://github.com/ElvisCuiHan/CSOMA/ and users can reproduce the results in the paper by running the *run_fractional.m* (in the folder High_Dim_D_Optimal_Example) and *run_glm_fisher.m* (in the folder Fractional_Polynomial_Regression_Example) files in Matlab, respectively.
